# M-polynomial driven machine learning models for predicting physicochemical properties of antibiotics

**DOI:** 10.1371/journal.pone.0338093

**Published:** 2025-12-11

**Authors:** Xin Li, Masoud Ghods, Negar Kheirkhahan, Jana Shafi

**Affiliations:** 1 Department of Gynecology, Renmin Hospital of Wuhan University, Wuhan, China; 2 Department of Applied Mathematics, Semnan University, Semnan-19111, Iran; 3 Department of Computer Engineering and Information, College of Engineering in Wadi Alddawasir, Prince Sattam Bin Abdulaziz University, Wadi Alddawasir, Saudi Arabia; Sepuluh Nopember Institute of Technology: Institut Teknologi Sepuluh Nopember, INDONESIA

## Abstract

Accurate prediction of the physicochemical properties of drug compounds is critical for the development of effective and safe antibiotics. In this study, we employ advanced machine learning techniques to address this challenge, using input data that includes M-Polynomials and various physicochemical descriptors. Three models were implemented: basic Support Vector Regression (SVR-Basic), optimized SVR (SVR-Tuned), and Random Forest (RF), trained on known compounds and tested on previously unseen drug samples to evaluate generalization.

Model performance was comprehensively assessed using R^2^, MSE, RMSE, and MAE, alongside detailed error and residual analyses to ensure precision and robustness. Furthermore, residual-based metrics such as the Mean Residual (MR), Standard Deviation of Residuals (Std Residual), and Interquartile Range (IQR) of Residuals were employed to provide complementary insights into prediction bias, consistency, and robustness.

By integrating feature importance analysis and ablation studies, the contribution of each molecular descriptor was systematically evaluated, providing deep insights into model stability and the key factors affecting predictive accuracy. Visual comparisons further illustrated the models’ behavior on training and test datasets.

The results demonstrate that the proposed approach not only improves predictive accuracy compared to prior studies but also offers a robust and reliable framework for real-world drug development. All models were implemented in Python 3.12.7, highlighting the practical applicability of machine learning in pharmaceutical research.

## Introduction

**Fig 1:**
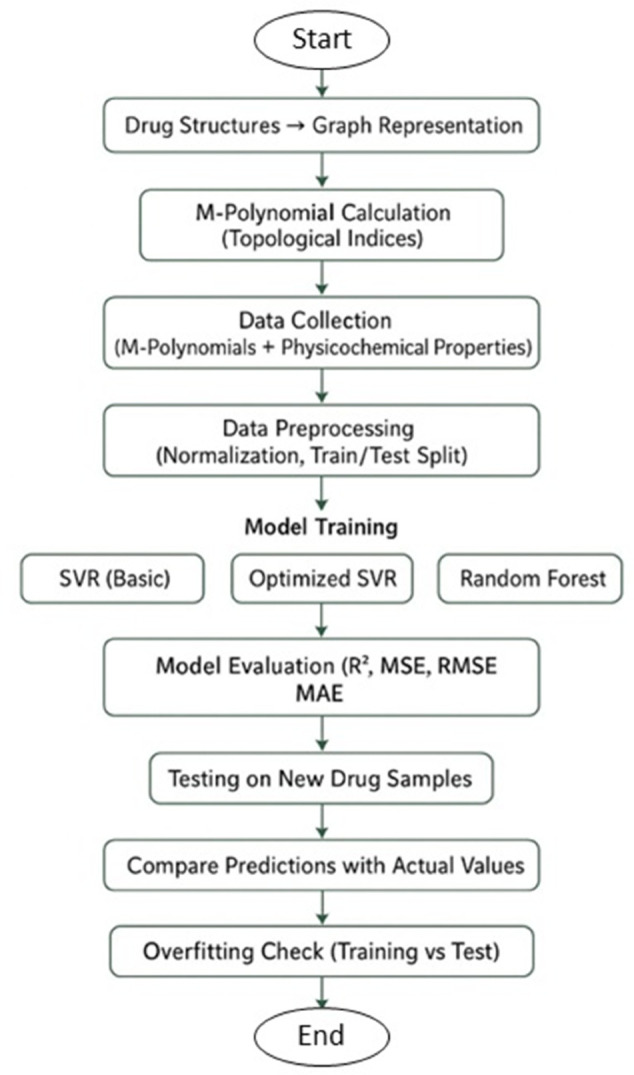
Diagram illustrating the workflow of the employed methodology. https://doi.org/10.6084/m9.figshare.30443927

Bacteria, commonly referred to as microorganisms or microbes, are widely present both within and around the human body. While certain bacterial species are essential for maintaining biological balance, others are responsible for infections such as pharyngitis and urinary tract infections [1]. Antibiotics are critical therapeutic agents used to inhibit or eliminate these pathogenic bacteria, playing a vital role in human medicine, veterinary care, and agriculture [2]. Despite their importance, accurately predicting the physicochemical properties of antibiotics remains a challenge, particularly for new or experimental drug candidates. Addressing this challenge is crucial because precise predictions can accelerate drug development and optimize therapeutic strategies. In recent years, computational approaches such as Quantitative Structure–Property Relationship (QSPR) modeling have emerged as powerful tools in drug discovery and design. QSPR methods correlate molecular structures with physicochemical properties using mathematical and statistical models, enabling the prediction of new compounds’ behavior without extensive experimental testing. One fundamental approach for examining the relationship between molecular properties and topological indices is QSPR modeling, which utilizes regression analysis to correlate physicochemical characteristics with topological descriptors. Similarly, QSAR models frequently incorporate these indices to predict biological activity [3,4]. A key concept in this framework is the molecular graph, where atoms are vertices and chemical bonds are edges, analyzed through chemical graph theory. Topological indices (TIs) are widely used descriptors in this context, capturing structural features that relate to molecular properties [5,6]. Several studies have applied TIs in QSPR and QSAR modeling. For instance, S. Kosari analyzed graph structures using the spectral radius and the Zagreb–Estrada index [7], while Kosari et al. proposed bounds for the KG-Sombor index and identified extremal trees achieving those bounds [8]. Beyond these studies, M-polynomials have been introduced as tools to calculate TIs more efficiently and to capture complex molecular features [9,10]. Prior research has shown their application in predicting drug properties for diverse therapeutic areas, including schizophrenia [11], anticancer drugs [12,13], and COVID-19 treatments [14,15]. Machine learning techniques, such as Basic SVR, Tuned SVR, and RF, have further enhanced the predictive power of QSPR models. These methods can capture nonlinear relationships that traditional regression models may overlook, improving prediction accuracy for complex datasets [16–18]. Previous works have demonstrated machine learning applications in QSPR modeling for anxiety treatment drugs and anti-tuberculosis medications [19]. In this study, we extend previous approaches by not only employing advanced machine learning models (SVR, Tuned SVR, and RF) but also focusing on experimental data and previously unseen drug samples used in the treatment of bacterial infections. This allows us to rigorously evaluate the models’ predictive accuracy and generalization in real-world settings. By combining mathematical modeling with clinical relevance, our study demonstrates that predictions are both theoretically robust and practically valuable, potentially guiding the development of new antibiotics and optimizing existing therapies. Furthermore, we examine the influence of temperature-related features and nonlinear topological indices on model performance, providing deeper insights into the factors that drive molecular behavior [20]. The methodological workflow is illustrated in Fig 1.

## Algorithm for predicting physicochemical properties of antibiotics drugs using machine learning models

**Step 1: Data Preparation and Preprocessing**

1. Collect data on M-Polynomials and physicochemical properties of drugs.
2. Handle missing values using median imputation.
3. Detect and remove outliers using the interquartile range (IQR) method.
4. Normalize features using Min–Max scaling to bring values into the range [0, 1].
5. Split the dataset into training (80)

**Step 2: Implementing Machine Learning Models**

1. Train the SVR-Basic model using the training data.
2. Optimize and train the SVR-Tuned model.
3. Train the RF model.

**Step 3: Evaluating Model Performance**

1. Calculate evaluation metrics including R^2^, MSE, RMSE, MAE, MR, Std, and IQR for each model.
2. Compare model predictions with actual values on the test data.
3. Assess the generalization ability of the models using new drug samples.

**Step 4: Preventing Overfitting**

1. Evaluate model performance on both training and test datasets.
2. Generate comparative plots to visualize model performance on both datasets.

**Step 5: Error and Residual Analysis**

1. Conduct error distribution analysis to evaluate prediction accuracy.
2. Generate residual plots to assess model consistency and identify patterns or biases.

**Step 6: Analyzing Results and Application in Drug Discovery**

1. Assess model accuracy and reliability. model.
2. Analyze the role of machine learning in drug development.
3. Emphasize the importance of using generalizable models for unseen data in real-world applications.

## Materials and methods

In this research, antibiotic drugs are represented as basic graph structures. To compute the topological indices of these drug molecules, methods like vertex partitioning, edge partitioning, and several computational techniques have been applied. Our analysis is confined to finite, simple, and connected graphs.

Let *G* denote a graph with a vertex set *V* and an edge set *E*. The degree of a vertex *u*, denoted as *d*_*u*_, is defined as the number of vertices adjacent to *u*.

Topological indices are important tools for analyzing molecular and graph structures, and the *M*-polynomial, introduced by Klavžar and Deutsch (2025), enables the calculation of degree-based indices [21]. The topological descriptors related to vertex degree that have been utilized are listed in Table 1.

**Table 1:** Characterization of topological descriptors.

Topological Descriptors	
M-Polynomial	$M(G;x,y)=\sum_{i\leq j}m_{ij}(G)x^{i}y^{j}$
First and Second Zagreb Indices	$M_{1}(G)=\sum_{uv\in E(G)}(d_{u}+d_{v}),\;M_{2}(G)=\sum_{uv\in E(G)}(d_{u}\cdot d_{v})$
Hyper-Zagreb Index	$HM(G)=\sum_{uv\in E(G)}(d_{u}+d_{v}{)}^{2}$
Randic Index	$R(G)=\sum_{uv\in E(G)}\sqrt{\frac{1}{d_{u}\cdot d_{v}}}$
Harmonic Index	$H(G)=\sum_{uv\in E(G)}\frac{2}{d_{u}+d_{v}}$
Sum-Connectivity Index	$S(G)=\sum_{uv\in E(G)}\sqrt{\frac{1}{d_{u}+d_{v}}}$
Forgotten Index	$F(G)=\sum_{uv\in E(G)}\left[(d_{u}{)}^{2}+(d_{v}{)}^{2}\right]$
Geometric-Arithmetic Index	$GA(G)=\sum_{uv\in E(G)}\frac{2\sqrt{d_{u}\cdot d_{v}}}{d_{u}+d_{v}}$
Atomic Bond Connectivity	$ABC(G)=\sum_{uv\in E(G)}\sqrt{\frac{d_{u}+d_{v}-2}{d_{u}\cdot d_{v}}}$

https://doi.org/10.6084/m9.figshare.29144426

## Methodology and analysis

**SVR-Basic**: Support Vector Regression (SVR) is a robust method for modeling nonlinear relationships by mapping input data into a higher-dimensional feature space using kernel functions such as linear, Gaussian (RBF), and polynomial. SVR minimizes prediction error within a specified tolerance (*ε*-tube), balancing model complexity through hyperparameters like *C* (penalty for errors) and *ε* (error tolerance).

**SVR-Tuned**: This variant enhances SVR performance by optimizing hyperparameters, including *C*, *γ* (gamma), and *ε*, using methods such as grid search and random sampling. Hyperparameter tuning allows the model to better capture the underlying data patterns and improve predictive accuracy.

**RF**: RF is an ensemble learning technique that constructs multiple decision trees using random subsets of both data points and features, then aggregates their predictions to enhance accuracy and reduce overfitting. Key hyperparameters include the number of estimators, maximum tree depth, and minimum samples per split. In addition, a brief feature importance analysis is performed for RF, which identifies the features that contribute most to the predictions. This analysis not only aids in interpreting the model’s behavior but also guides feature selection for future studies. The performance of all models was evaluated using Mean Squared Error (MSE), Root Mean Squared Error (RMSE), Mean Absolute Error (MAE), R^2^ score, Mean Residual (MR), Standard Deviation of Residuals (Std), and Interquartile Range (IQR) of Residuals to provide a comprehensive assessment of predictive accuracy.

### Assessment metrics

To evaluate prediction accuracy, four key metrics (models 1 to 7 ) are employed. These metrics measure the precision and efficiency of each model.

$$MSE=\underset{\text{Mean Squared Error}}{\underbrace{\frac{1}{n}\sum_{i=1}^{n}(y_{i}-\hat{y_{i}}{)}^{2}}}$$ (1)

$$RMSE=\underset{\text{Root Mean Squared Error}}{\underbrace{\sqrt{\frac{1}{n}\sum_{i=1}^{n}(y_{i}-\hat{y_{i}}{)}^{2}}}}$$ (2)

$$MAE=\underset{\text{Mean Absolute Error}}{\underbrace{\frac{1}{n}\sum_{i=1}^{n}|y_{i}-\hat{y_{i}}|}}$$ (3)

Where *y*_*i*_ is the actual value, $\hat{y_{i}}$ is the predicted value, and *n* is the number of samples.

$$R^{2}=\underset{\text{Coefficient of Determination}}{\underbrace{1-\frac{\sum_{i=1}^{n}(y_{i}-\hat{y_{i}}{)}^{2}}{\sum_{i=1}^{n}(y_{i}-\bar{y_{i}}{)}^{2}}}}$$ (4)

Where $\bar{y}$ is the mean of actual values.

$$MR=\underset{\text{Mean Residual (MR)}}{\underbrace{\frac{1}{n}\sum_{i=1}^{n}(y_{i}-\hat{y_{i}})}}$$ (5)

$$Std=\underset{\text{Standard Deviation of Residuals}}{\underbrace{\sqrt{\frac{1}{n-1}\sum_{i=1}^{n}{\left[(y_{i}-\hat{y_{i}})-MR\right]}^{2}}}}$$ (6)

$$IQR=\underset{\text{Interquartile Range}}{\underbrace{Q_{3}-Q_{1}}}$$ (7)

The best model is the one where the coefficient of determination (*R*^2^) is close to 1, as this indicates high accuracy in explaining the variance in the data. Additionally, the MSE, RMSE, and MAE should be as close to 0 as possible, indicating lower error and better performance. Furthermore, residual-based metrics such as the Mean Residual (MR), Standard Deviation of Residuals (Std Residual), and Interquartile Range (IQR) of Residuals provide complementary information about prediction bias, consistency, and robustness. Among the three evaluated models SVR-Basic, SVR-Tuned, and RF a model with *R*^2^ close to 1, low error metrics, and low residual variability (small Std Residual and IQR) provides the most accurate and reliable predictions.

### Analysis and comparison of the performance of machine learning models in QSPR evaluation

In this section, the predictive performance of three machine learning models across five physicochemical properties of antibiotic compounds is evaluated. The analysis includes both numerical metrics and visual assessments to provide a comprehensive understanding of model accuracy and reliability. The models evaluated are **Basic Support Vector Regression (SVR-Basic)**, **Tuned Support Vector Regression (SVR-Tuned)**, and **Random Forest (RF)**.

Nineteen drug compounds were analyzed, and the calculated topological indices are provided in Supplementary Table S1 (S1 Table). Experimental values for the physicochemical properties, sourced from [22], along with predicted values generated using Python, are summarized in various tables. In the main article, Table 2 presents the predicted COM and MR properties, while the remaining properties are available through Supplementary Tables S2 and S3 (S2 Table, S3 Table).

**Table 2:** Prediction results of machine learning models.

Comparative Analysis of Machine Learning Models								
**Chemical Formulas**	**Actual COM**	**SVR-Basic**	**SVR-Tuned**	**Random-Forest**	**Actual MR**	**SVR-Basic**	**SVR-Tuned**	**Random-Forest**
$C_{10}H_{11}N_{3}O_{3}S$	346	815.021	345.255	572.320	62.5	113.655	62.545	85.724
$C_{12}H_{17}N_{3}O_{4}S$	491	813.374	489.094	572.320	72.7	112.072	72.582	85.724
$C_{16}H_{19}N_{3}O_{4}S$	562	811.647	562.545	567.420	83.3	111.079	83.311	85.474
$C_{17}H_{18}FN_{3}O_{3}$	571	811.641	570.965	571.910	89.4	111.048	89.219	89.420
$C_{16}H_{19}N_{3}O_{5}S$	590	811.677	590.011	593.280	89.9	111.084	90.001	89.537
$C_{16}H_{17}N_{3}O_{4}S$	600	811.729	598.272	588.860	91.1	111.359	91.039	90.542
$C_{18}H_{37}N_{5}O_{9}$	609	813.666	610.635	642.380	91.5	111.089	91.497	90.667
$C_{18}H_{20}FN_{3}O_{4}$	634	812.021	634.255	633.740	96.8	111.524	96.841	96.822
$C_{21}H_{43}N_{5}O_{7}$	636	814.610	638.490	682.530	101.8	113.254	102.007	109.748
$C_{17}H_{25}N_{3}O_{5}S$	679	812.075	677.997	657.060	109.0	115.386	108.990	111.078
$C_{21}H_{24}FN_{3}O_{4}$	727	814.128	727.602	661.320	111.7	113.392	111.713	112.014
$C_{22}H_{43}N_{5}O_{13}$	819	819.000	819.009	854.180	116.0	116.000	116.010	116.267
$C_{21}H_{39}N_{7}O_{12}$	940	820.109	938.337	942.100	121.0	117.132	121.064	121.958
$C_{22}H_{24}N_{2}O_{8}$	956	818.397	956.010	945.370	122.6	114.793	122.590	119.979
$C_{23}H_{27}N_{3}O_{7}$	971	818.824	970.372	951.370	134.9	117.559	134.890	131.051
$C_{38}H_{72}N_{2}O_{12}$	1150	821.639	1150.220	1154.400	151.3	119.114	151.242	151.101
$C_{37}H_{67}NO_{13}$	1180	821.590	1179.750	1170.300	189.2	119.964	189.211	188.692
$C_{38}H_{69}NO_{13}$	1190	821.454	1190.011	1177.400	194.0	119.820	194.023	191.632
$C_{29}H_{39}N_{5}O_{8}$	1240	821.661	1236.959	1180.890	197.6	119.836	197.456	193.108

https://doi.org/10.6084/m9.figshare.29144432

**Fig 2:**
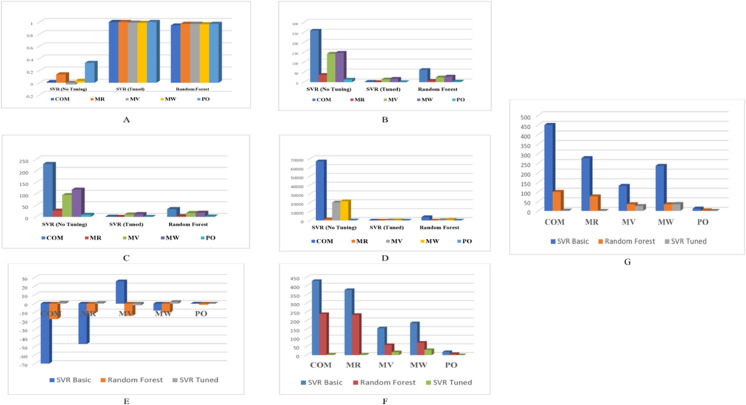
Comparison of machine learning models (basic SVR, tuned SVR, and RF) in predicting physicochemical properties of antibiotic drugs. R^2^, MSE, MAE, RMSE, MR, Std, and IQR are shown for training and test datasets to illustrate model accuracy and generalization. **A**: R^2^ comparison of ML models for predicting drug properties. **B**: RMSE comparison of ML models for predicting drugs. **C**: MAE comparison of ML models for predicting drug properties. **D**: MSE comparison of ML models for predicting drug properties. **E**: Mean residual (MR) comparison of ML models for predicting. **F**: Standard deviation (Std) of residuals comparison of ML models for predicting. **G**: Interquartile range (IQR) of residuals comparison of ML models for predicting. Source: DOI:10.6084/m9.figshare.29144435

These predictions were compared against experimental data to assess model accuracy. Quantitative metrics such as R^2^, MSE, MAE, RMSE, MR, standard deviation (Std), and interquartile range (IQR) are reported in Tables 3, 4, 5, 6, 7, 8, 9. Additionally, a visual comparison of model performance using selected metrics is shown in Fig 2. The machine learning algorithms used for these predictions are described in detail in the following sections.

**Table 3:** Analysis of the performance of machine learning models for drug property prediction based on the R^2^ metric.

Models	COM	MR	MV	MW	PO
SVR-Basic	0.011136238	0.138954133	-0.03031247	0.032089992	0.326599397
SVR-Tuned	0.999976452	0.999995247	0.990169642	0.987028108	0.999995603
Random-Forest	0.944373630	0.971139058	0.971782379	0.964930471	0.970835086

https://doi.org/10.6084/m9.figshare.29145902

**Table 4:** Analysis of the performance of advanced ML models for drug property prediction based on the MSE metric.

Models	COM	MR	MV	MW	PO
SVR-Basic	66971.9789	1305.151429	20299.08454	21632.11019	160.2979211
SVR-Tuned	1.594848005	0.007204806	193.6764467	289.9126973	0.001046725
Random-Forest	3767.362342	43.74668242	555.9399589	783.7793973	6.942487158

https://doi.org/10.6084/m9.figshare.29145905

**Table 5:** Analysis of the performance of advanced ML models for drug property prediction based on the RMSA metric.

Models	COM	MR	MV	MW	PO
SVR-Basic	258.789449	36.12687959	142.4748558	147.0785851	12.66088153
SVR-Tuned	1.262872917	0.084881128	13.91676854	17.02682288	0.03235313
Random-Forest	61.37884279	6.614127488	23.57837906	27.99606039	2.634859988

https://doi.org/10.6084/m9.figshare.29145908

**Table 6:** Analysis of the performance of advanced ML models for drug property prediction based on the MAE metric.

Models	COM	MR	MV	MW	PO
SVR-Basic	230.5219014	27.24332899	94.93726578	118.7155829	8.939168797
SVR-Tuned	0.883530924	0.058963294	10.642956	12.37942743	0.02324393
Random-Forest	34.18578947	3.464210526	16.73752632	18.12536842	1.523789474

https://doi.org/10.6084/m9.figshare.29145911

**Table 7:** Analysis of the performance of advanced ML models for drug property prediction based on the mean residual metric.

Models	COM	MR	MV	MW	PO
SVR-Basic	−69.6785	−46.906	25.64194	−8.12174	0.081604
SVR-Tuned	0.982566	0.805262	−2.07434	1.792562	−0.00106
Random-Forest	−17.8576	−11.171	−14.1765	−10.3209	−1.31538

https://doi.org/10.6084/m9.figshare.30011146

**Table 8:** Analysis of the performance of advanced ML models for drug property prediction based on the std residual metric.

Models	COM	MR	MV	MW	PO
SVR-Basic	428.885818	377.0055068	155.7821455	185.2197339	18.00600838
SVR-Tuned	3.159584389	3.038742283	17.08657027	30.14049248	0.063035687
Random-Forest	238.6656373	233.7425558	58.53799807	72.4473699	7.159355684

https://doi.org/10.6084/m9.figshare.30011353

**Table 9:** Analysis of the performance of advanced ML models for drug property prediction based on the IQR residual metric.

Models	COM	MR	MV	MW	PO
SVR-Basic	450.3544945	276.6445317	131.3471582	236.5366099	12.0964274
SVR-Tuned	1.4527288	0.181922794	25.66632321	35.83517118	0.037285217
Random-Forest	99.66	76.038	34.95	34.7054	2.323

https://doi.org/10.6084/m9.figshare.30011356

The results indicate that parameter tuning of the SVR model significantly improves its performance across all evaluation metrics, achieving higher *R*^2^ values and lower error rates. While the basic SVR shows weak predictive ability and the Random Forest (RF) performs reasonably well, the tuned SVR consistently outperforms the other models in predicting drug properties.

In Supplementary Table S4 (S4 Table), a simple comparison with a baseline model (e.g., Linear Regression) has been added to provide context for model performance. Data and supplementary materials are available at the following sources:

### Performance analysis of machine learning models using error distributions and residual plots

In this section, we provide a detailed examination of the models’ error distributions and residual patterns. These analyses complement the overall performance evaluation presented in the previous section and help to identify the stability and reliability of each model in predicting different physicochemical properties.

**Fig 3:**
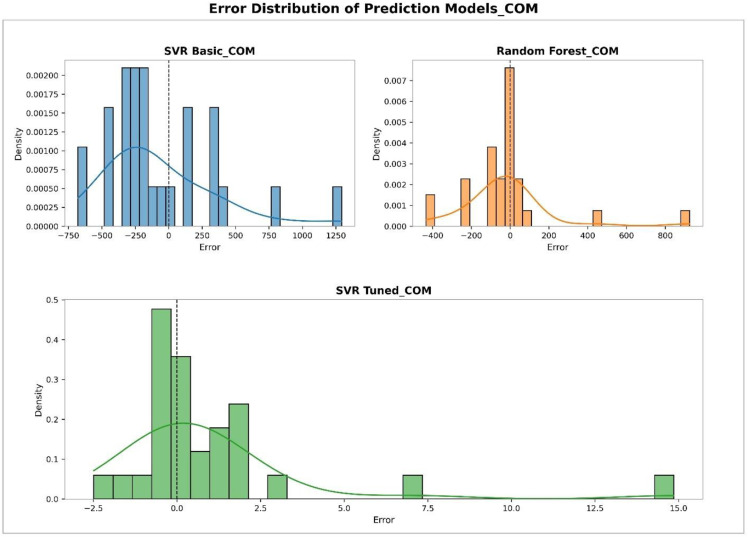
Error distributions of the models in predicting COM. Histograms and KDE plots display the variability and precision of predictions for clear comparison. DOI:10.6084/m9.figshare.29143448

**Fig 4:**
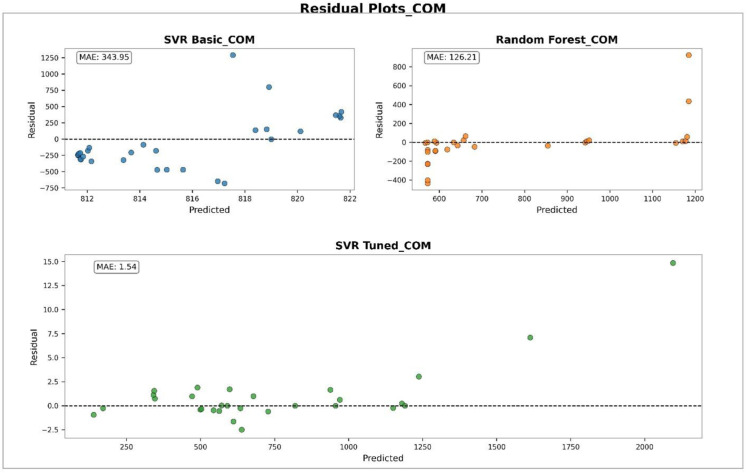
Comparison of residual distributions for different models in predicting COM. DOI:10.6084/m9.figshare.29143457

**Fig 5:**
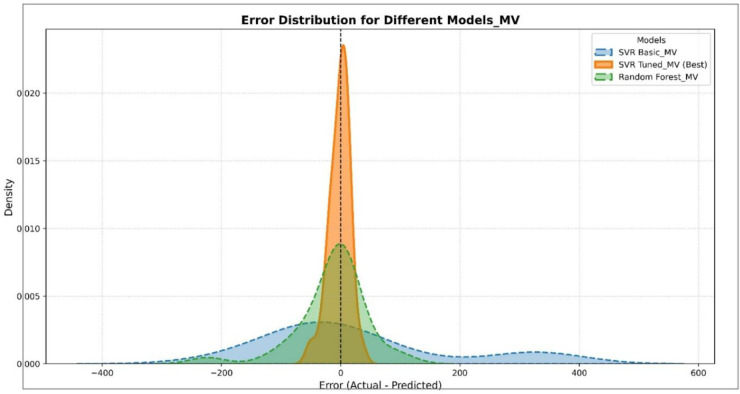
Error distribution of different models in predicting MV. DOI:10.6084/m9.figshare.29143463

**Fig 6:**
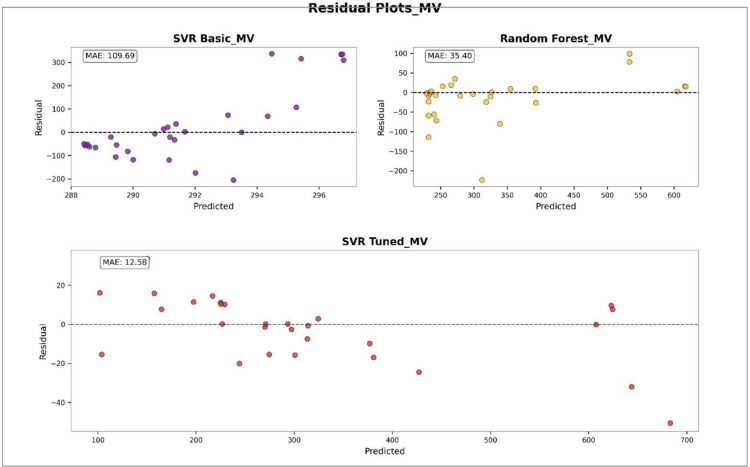
Residual plot of different models in predicting MV. DOI:10.6084/m9.figshare.29143472

To evaluate the performance of different models in predicting physicochemical properties, both error distribution analyses and residual plots were conducted for various features. Fig 3 illustrates the error distribution of different models in predicting the COM property. This figure was generated using histograms combined with Kernel Density Estimation (KDE) curves. The Tuned SVR model shows a narrow and symmetric error distribution centered around zero, indicating high prediction accuracy and low variance. The RF model demonstrates moderate performance with a slightly wider error spread, while the Basic SVR model exhibits the widest error range and the least concentration around zero, reflecting the weakest predictive performance. Fig 4 presents the residual plots of different models for predicting COM. In this plot, residuals are displayed against the predicted values to identify error patterns and potential instabilities. The Tuned SVR model again exhibits a stable and unbiased pattern with residuals evenly dispersed around zero. The RF model follows with moderate stability, while the Basic SVR shows a scattered and less symmetrical distribution of residuals, indicating less reliable predictions. Fig 5 shows the error distribution for the prediction of the MV property. Similar to the observations for COM, the Tuned SVR model achieves superior performance with a sharply peaked distribution near zero. The RF model demonstrates intermediate accuracy, while the Basic SVR again shows broader error dispersion, indicating inferior prediction accuracy. Fig 6 presents the residual plot for MV, which further confirms the trends observed in error distributions. The Tuned SVR maintains a tight and balanced spread of residuals around zero, underscoring its robustness and consistency. The RF model shows slightly greater residual spread but remains reasonably stable. In contrast, the Basic SVR model exhibits high variability and irregular residual patterns, signifying poor stability and less accurate predictions. Overall, these analyses consistently indicate that the Tuned SVR model outperforms the others, providing the most accurate and stable predictions across both COM and MV properties. The RF model ranks second, offering acceptable performance, while the Basic SVR model consistently shows the weakest predictive capacity.

### Evaluation of algorithms on test data

In this section, we examine the predictive performance of the three machine learning algorithms on previously unseen test data. This analysis complements the training evaluation and helps assess the models’ generalization capability and reliability when applied to new drug compounds.

As mentioned in the previous section, data from nineteen different types of drugs were initially used to train the three machine learning algorithms in Python, aiming to predict their physicochemical properties. Subsequently, ten drug samples were introduced as test data to evaluate model performance.

The comparative results of these predictions for the COM and MR properties are presented as examples in Table 10, while the remaining properties are provided in Supplementary Tables S5 and S6 (S5 Table, S6 Table).

**Table 10:** Comparison of Actual and Predicted COM and MR Values for Test Drug Samples.

Chemical Formula	Actual COM	SVR-Basic	SVR-Tuned	RF	Actual MR	SVR-Basic	SVR-Tuned	RF
C_18_H_33_ClN_2_O_5_S	138	817.2242816	138.9393992	572.32	25.4	116.0175305	25.62722195	85.724
C_18_H_34_N_2_O_6_S	170	816.9597658	170.2459142	572.32	41	115.5912842	40.91436594	85.724
C_16_H_20_FN_3_O_4_	342	814.6535412	340.8702735	572.32	58.9	114.1633545	59.0159242	85.724
C_48_H_62_N_4_O_12_	345	815.6395338	343.4338781	572.32	72.6	113.1276373	72.59749192	85.724
C_46_H_62_N_4_O_11_	472	812.1512102	471.0098558	572.32	83	111.4481342	83.09562374	88.26
C_8_H_13_N_3_O_4_S	499	811.7516298	499.3900652	590.52	93.6	111.4970079	93.54888331	90.742
C_6_H_9_N_3_O_3_	502	811.7433596	502.3230023	590.52	104.7	111.960682	104.6144393	101.881
C_11_H_12_Cl_2_N_2_O_5_	543	811.8377586	543.462634	618.48	107.9	112.1718367	107.875109	103.963
C_17_H_15_FN_6_O_3_	1620	818.9165589	1612.898341	1184.8	213.1	117.5990481	212.9531734	188.686
C_3_H_7_O_4_P	2110	817.5356784	2095.145291	1184.8	222.9	116.3854701	222.8424053	189.67

DOI: 10.6084/m9.figshare.29145917

To evaluate the predictive performance of the proposed machine learning models on the test data, several statistical metrics, including R^2^, MSE, RMSE, and MAE, were employed. These metrics provide a comprehensive assessment of both the accuracy and robustness of the models. The comparative results for these metrics are presented individually in Supplementary Tables S7, S8, S9, and S10 (S7 Table, S8 Table, S9 Table, S10 Table), enabling a direct and detailed comparison of the models’ predictive capabilities.

**Fig 7:**
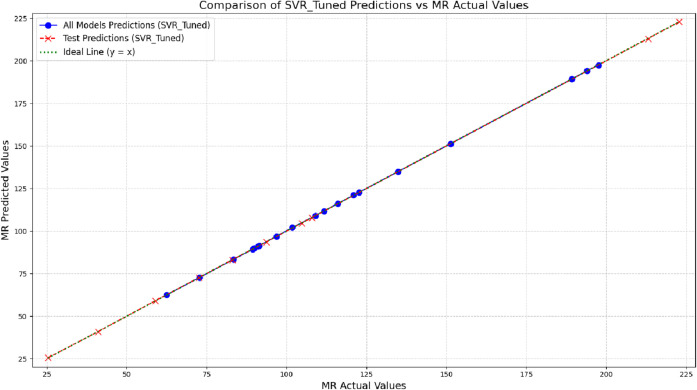
Visual comparison of predicted and actual MR values by SVR-Tuned across training and test sets. DOI:10.6084/m9.figshare.29143475

**Fig 8:**
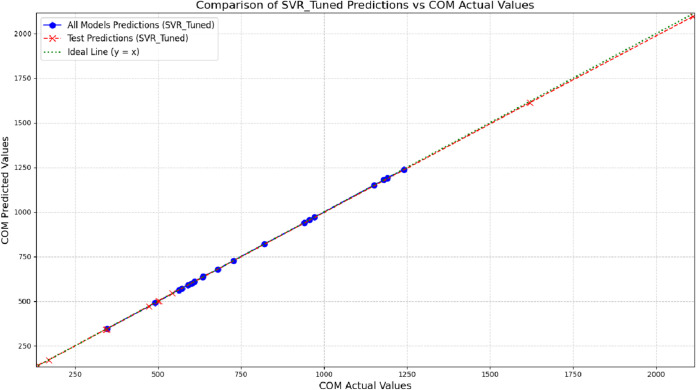
Comparison of predicted and actual COM values using SVR-tuned model on training and test data. DOI:10.6084/m9.figshare.29143523

**Fig 9:**
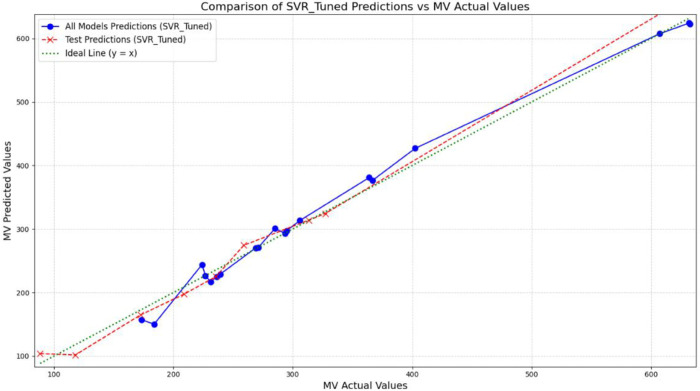
Comparison of SVR-tuned predictions and actual MV values for training and test sets. DOI:10.6084/m9.figshare.29143526

**Fig 10:**
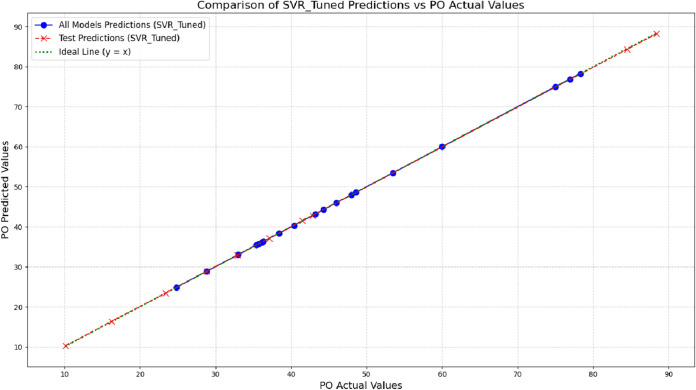
Comparison of SVR-tuned predictions and actual PO values for training and test sets. DOI:10.6084/m9.figshare.29143529

Another notable strength of the proposed approach is the model’s stability across the entire range of investigated properties. The performance of the ten test samples is illustrated in Figs 7, 8, 9, and 10, providing a visual analysis of each algorithm’s accuracy and reliability in predicting drug properties. The results indicate that the models demonstrated consistent performance on both training and test datasets, implying that they effectively generalized to new data while maintaining high accuracy and robustness. To provide a more concrete representation of the model’s performance, three different algorithms were evaluated across four physicochemical properties: MV, PO, COM, and MR. The close alignment of the data points (blue representing all data and red representing test data) with the ideal line (*y* = *x*) illustrates the strong predictive capability of the models. The substantial overlap between training and test predictions indicates consistent performance on unseen data, underscoring strong generalization ability. Moreover, the absence of significant deviations from the ideal line suggests low prediction error and effective learning of the underlying physicochemical patterns. Another notable strength of the proposed approach is the model’s stability across the entire range of investigated properties.

## Feature importance

**Fig 11:**
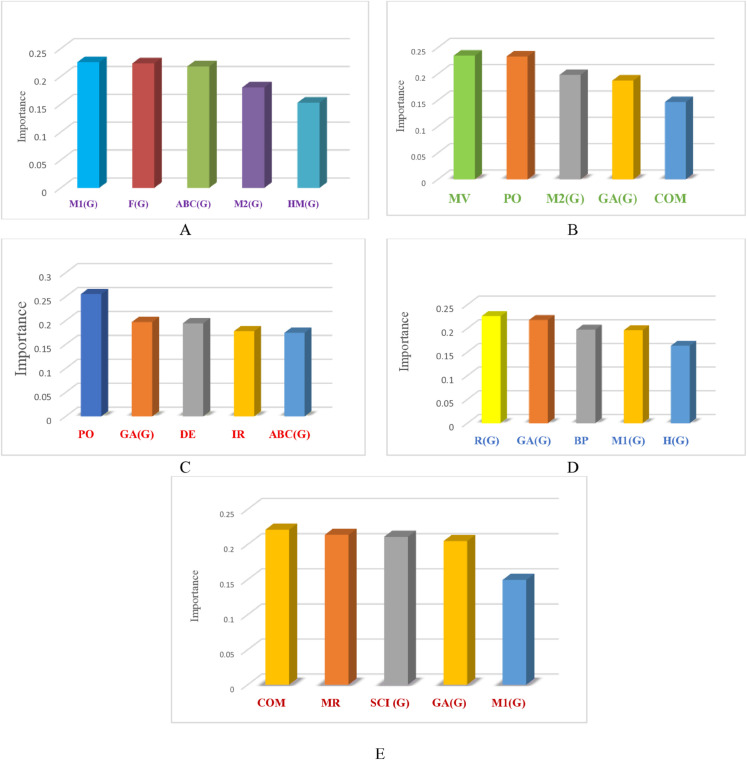
Feature importance in predicting different indicators (COM, MR, MV, MW, PO). **A**: Feature importance for predicting COM. **B**: Feature importance for predicting MR. **C**: Feature importance for predicting MV. **D**: Feature importance for predicting MW. **E**: Feature importance for predicting PO. DOI:10.6084/m9.figshare.30068917

In this section, we present a systematic analysis of feature importance to identify the key molecular descriptors influencing the prediction of five chemical properties. This analysis helps to understand which features contribute most to model accuracy and provides insight into the relative impact of each descriptor on predictive performance. To predict five chemical properties, including COM, MR, PO, MW, and MV, a systematic workflow was followed. First, data cleaning and preparation were conducted: data were imported from an Excel file, duplicate or unnecessary columns were removed, and textual values, such as numbers containing commas, were converted to numeric types. Subsequently, in the feature selection and target variable stage, the values of each property were separated as the target variable, while the remaining descriptors were chosen as input features. The dataset was then split into training (80%) and testing (20%) sets (Train-Test Split). To improve model convergence and standardize the scale of the variables, feature standardization was applied. Following this, Recursive Feature Elimination (RFE) in combination with the RF algorithm was used to select the five most important features for each target property. In the model training phase, three models were employed: Support Vector Regression without hyperparameter tuning (SVR Basic), Support Vector Regression with hyperparameter tuning (SVR Tuned), and RF. Model evaluation was performed for both training and testing sets using four metrics: MAE, RMSE, MSE, and R^2^. To further analyze the results, feature importance tables and plots were generated. Specifically, Table 11 presents the comparison of feature importance across different predictive models for COM, MR, MV, MW, and PO, while Fig 11 illustrates the relative importance of features in predicting these chemical indices.

**Table 11:** Comparison of feature importance across different prediction models (COM, MR, MV, MW, PO).

Fe COM	Im COM	Fe MR	Im MR	Fe MV	Im MV	Fe MW	Im MW	Fe PO	Im PO
M1(G)	0.2255	MV	0.23464	PO	0.25543	R(G)	0.22575	COM	0.220960
F(G)	0.2234	PO	0.23304	GA(G)	0.19725	GA(G)	0.21759	MR	0.21368
ABC(G)	0.2179	M2(G)	0.19809	DE	0.19458	BP	0.19732	SCI(G)	0.210926
M2(G)	0.1802	GA(G)	0.18747	IR	0.17795	M1(G)	0.19601	GA(G)	0.20473
HM(G)	0.1527	COM.1	0.14675	ABC(G)	0.174779	H(G)	0.16331	M1(G)	0.149698

DOI: 10.6084/m9.figshare.30016408

## Ablation study

In this section, we investigate the contribution of individual features to model performance through a systematic ablation study. This analysis quantifies the impact of each molecular descriptor and evaluates model robustness when specific features are excluded.

To examine the contribution of individual features in more detail, an ablation study was conducted. In this study, models were systematically trained and evaluated after removing specific features or groups of features. The results reveal the relative importance of each feature and show how model accuracy is affected when certain descriptors are excluded.

**Fig 12:**
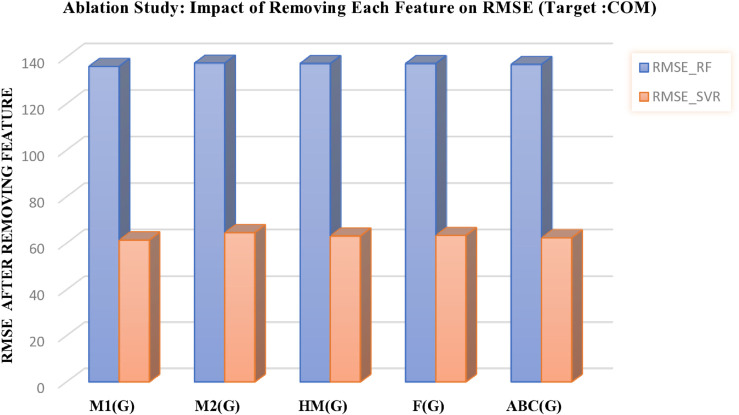
Ablation analysis of features with respect to RMSE (target: COM). DOI:10.6084/m9.figshare.30016429

**Fig 13:**
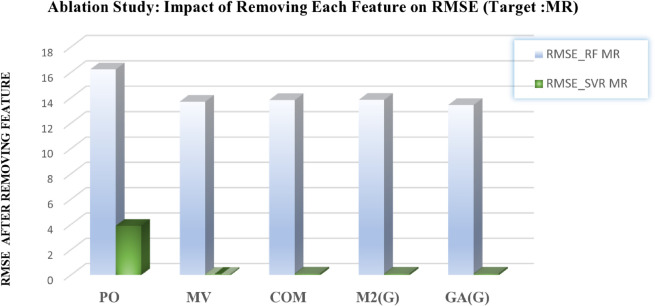
Ablation analysis of features with respect to RMSE (target: MR). DOI:10.6084/m9.figshare.30016432

**Fig 14:**
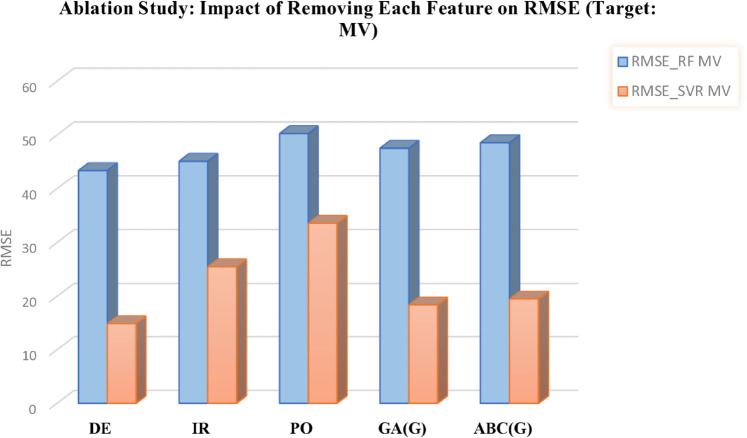
Ablation analysis of features with respect to RMSE (target: MV). DOI:10.6084/m9.figshare.30016435

**Fig 15:**
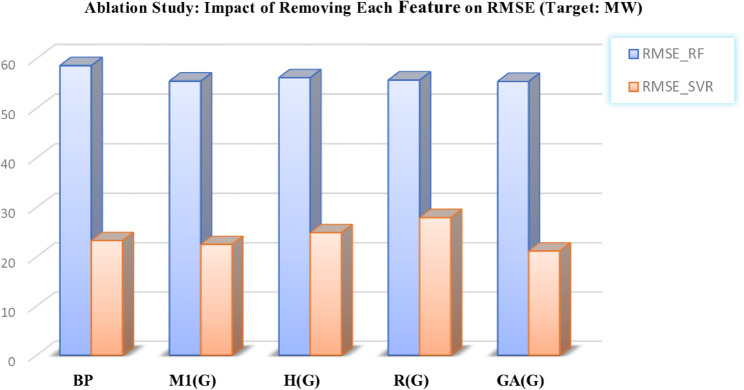
Ablation analysis of features with respect to RMSE (target: MW). DOI:10.6084/m9.figshare.30016438

**Fig 16:**
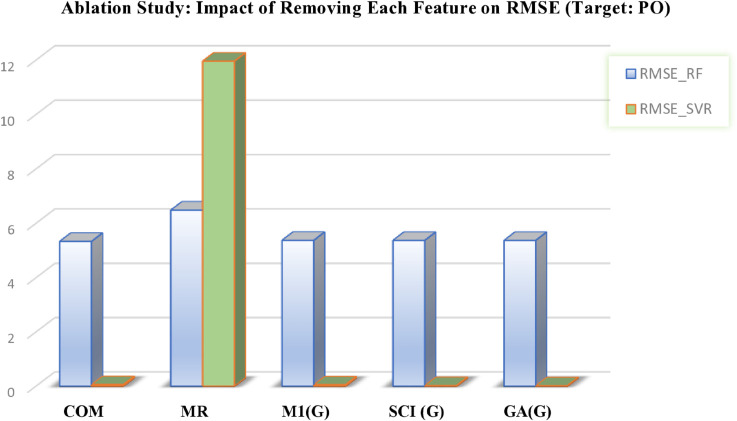
Ablation analysis of features with respect to RMSE (target: PO). DOI:10.6084/m9.figshare.30016444

This analysis provides deeper insights into the robustness of the predictive models and highlights the critical role of selected features in forecasting chemical properties. The outcomes are presented in Tables 12–13 and in Figs 12, 13, 14, 15, and 16, which clearly illustrate how the exclusion of each feature influences model performance and identify the most important features contributing to prediction accuracy.

**Table 12:** Ablation study results: Impact of feature removal on RMSE for COM and MR targets using RF and SVR models.

Feature Removed COM	RMSE-RF COM	RMSE-SVR COM	Feature Removed MR	RMSE-RF MR	RMSE-SVR MR
M1(G)	136.0237	61.3839	PO	16.2335	3.9021
M2(G)	137.4284	64.5710	MV	13.7033	0.0955
HM(G)	137.2593	63.1132	COM	13.8259	0.1215
F(G)	137.2593	63.3622	M2(G)	13.8429	0.1148
ABC(G)	136.8545	62.3282	GA(G)	13.4415	0.1169

DOI: 10.6084/m9.figshare.30016411

**Table 13:** Ablation study results: Impact of feature removal (FR) on RMSE for MV, MW, and PO targets using RF and SVR models.

FR MV	RMSE-RF MV	RMSE-SVR MV	FR MW	RMSE-RF MW	RMSE-SVR MW	FR PO	RMSE-RF PO	RMSE-SVR PO
DE	43.4112	14.8902	BP	58.5824	23.2947	COM	5.3374	0.0587
IR	45.1303	25.5183	M1(G)	55.4591	22.5085	MR	6.4896	11.9640
PO	50.2825	33.6023	H(G)	56.1515	24.8892	M1(G)	5.3767	0.0490
GA(G)	47.5546	18.4053	R(G)	55.6691	27.9348	SCI(G)	5.3747	0.0239
ABC(G)	48.5848	19.5190	GA(G)	55.3994	21.1827	GA(G)	5.3747	0.0185

DOI: 10.6084/m9.figshare.30016417

## Conclusions

This study demonstrates the key role of advanced machine learning in accurately predicting the physicochemical properties of drug compounds, which is an important step toward accelerating antibiotic development. Among the evaluated models, SVR-Tuned consistently demonstrated superior performance, achieving substantially higher predictive accuracy and robustness compared to SVR-Basic and RF. Error and residual analyses confirmed the stability of the proposed framework, and evaluations were conducted on both training and unseen test data, clearly demonstrating the models’ generalization capability. In addition, feature importance analysis and an ablation study were performed to investigate the contribution of individual molecular descriptors to prediction accuracy. In the feature importance analysis, after data cleaning, preprocessing, and feature scaling, Recursive Feature Elimination (RFE) combined with the RF algorithm was applied to identify the most important descriptors for each target property (COM, MR, MV, MW, and PO). The results indicated that descriptors such as M1(G), PO, GA(G), and COM played a crucial role in predicting various chemical indices, enhancing model interpretability and highlighting the chemical and biological relevance of key features. The ablation study further examined the impact of systematically removing specific features or groups of features on model performance. The exclusion of key descriptors (e.g., M1(G), PO, GA(G), and DE) resulted in a noticeable increase in errors, particularly for SVR-Tuned, which otherwise exhibited the best overall performance. These analyses demonstrated that model accuracy depends not only on algorithmic optimization but also on the careful and meaningful selection of molecular descriptors.

Overall, these findings establish SVR-Tuned as a highly effective, robust, and reliable model for drug property prediction. Feature importance analysis and the ablation study provide deep insights into the contribution of molecular descriptors and the stability of the models, while evaluations on test data confirm strong generalization ability, offering a solid foundation for future applications in computational drug discovery and pharmaceutical research.

The code is available in Supplementary Appendix S1 (S1 Appendix) or via the DOI: Code for predicting physicochemical properties using SVR-Basic, SVR-Tuned, and RF.

## Supporting information

S1 Table Supplementary Table 1. Available at: https://doi.org/10.6084/m9.figshare.30069574.
(PDF)

S2 Table Supplementary Table 2. Available at: https://doi.org/10.6084/m9.figshare.30069577.
(PDF)

S3 Table Supplementary Table 3. Available at: https://doi.org/10.6084/m9.figshare.30069580.
(PDF)

S4 Table Supplementary Table 4. Available at: https://doi.org/10.6084/m9.figshare.30069583.
(PDF)

S5 Table Supplementary Table 5. Available at: https://doi.org/10.6084/m9.figshare.30069586.
(PDF)

S6 Table Supplementary Table 6. Available at: https://doi.org/10.6084/m9.figshare.30069589.
(PDF)

S7 Table Supplementary Table 7. Available at: https://doi.org/10.6084/m9.figshare.30069598.
(PDF)

S8 Table Supplementary Table 8. Available at: https://doi.org/10.6084/m9.figshare.30069604.
(PDF)

S9 Table Supplementary Table 9. Available at: https://doi.org/10.6084/m9.figshare.30069607.
(PDF)

S10 Table Supplementary Table 10. Available at: https://doi.org/10.6084/m9.figshare.30069610.
(PDF)

S1 Appendix Supplementary Appendix 1. Python code for predicting physicochemical properties using SVR-Basic, SVR-Tuned, and RF. Available at: https://doi.org/10.6084/m9.figshare.28790726.
(PY)

## References

[1] Medical News Today. What to know about infections?; 2021. https://www.medicalnewstoday.com/articles/196271

[2] U S NL of M. Antibiotics. https://medlineplus.gov/antibiotics.html

[3] Havare ÖÇ. Quantitative structure analysis of some molecules in drugs used in the treatment of COVID-19 with topological indices. Polycycl Aromat Compd. 2021;42(8):5249–60. doi: 10.1080/10406638.2021.1934045

[4] Zaman S, Mushtaq M, Danish M, Ali P, Rasheed S. Topological characterization of some new anti-viral drugs for cancer treatment. BioNanoSci. 2024;14(5):4864–76. doi: 10.1007/s12668-024-01500-2

[5] Ghorbani M, Hosseinzadeh M A. A new version of Zagreb indices. Filomat. 2012;26(1):93–100. doi: 10.2298/fil1201093g

[6] Veličković P. Everything is connected: Graph neural networks. Curr Opin Struct Biol. 2023;79:102538. doi: 10.1016/j.sbi.2023.102538 36764042

[7] Kosari S. On spectral radius and Zagreb Estrada index of graphs. Asian-European J Math. 2023;16(10). doi: 10.1142/s1793557123501760

[8] Kosari S, Dehgardi N, Khan A. Lower bound on the KG-Sombor index. Commun Comb Optim. 2023;8:751–7. doi: 10.22049/cco.2023.28666.1662

[9] Hasani M, Ghods M. Calculation of topological indices along with MATLAB coding in QSPR analysis of calcium channel-blocking cardiac drugs. J Math Chem. 2024;62(10):2456–77. doi: 10.1007/s10910-023-01570-9

[10] Alali AS, Ali S, Hassan N, Mahnashi AM, Shang Y, Assiry A. Algebraic structure graphs over the commutative ring Zm: Exploring topological indices and entropies using M-polynomials. Mathematics. 2023;11(18):3833. doi: 10.3390/math11183833

[11] Zhang X, Saif MJ, Idrees N, Kanwal S, Parveen S, Saeed F. QSPR analysis of drugs for treatment of schizophrenia using topological indices. ACS Omega. 2023;8(44):41417–26. doi: 10.1021/acsomega.3c05000 37970009 PMC10633864

[12] Shi X, Cai R, Ramezani Tousi J, Talebi AA. Quantitative structure–property relationship analysis in molecular graphs of some anticancer drugs with temperature indices approach. Mathematics. 2024;12(13):1953. doi: 10.3390/math12131953

[13] Zhang Y, Khalid A, Siddiqui MK, Rehman H, Ishtiaq M, Cancan M. On analysis of temperature based topological indices of some covid-19 drugs. Polycycl Aromat Compd. 2022;43(4):3810–26. doi: 10.1080/10406638.2022.2080238

[14] Jahanbani A, Khoeilar R, Cancan M. [Retracted] On the temperature indices of molecular structures of some networks. J Math. 2022;2022(1). doi: 10.1155/2022/4840774

[15] Tamilarasi W, Balamurugan BJ. QSPR and QSTR analysis to explore pharmacokinetic and toxicity properties of antifungal drugs through topological descriptors. Sci Rep. 2025;15(1):18020. doi: 10.1038/s41598-025-01522-0 40410226 PMC12102203

[16] Bargam B, Boudhar A, Kinnard C, Bouamri H, Nifa K, Chehbouni A. Evaluation of the support vector regression (SVR) and the random forest (RF) models accuracy for streamflow prediction under a data-scarce basin in Morocco. Discov Appl Sci. 2024;6(6). doi: 10.1007/s42452-024-05994-z

[17] Breiman L. Random forests. Mach Learn. 2001;45(1):5–32. doi: 10.1023/a:1010933404324

[18] Pashankar SS, Shendage JD, Pawar DrJ. Machine learning techniques for stock price prediction – A comparative analysis of linear regression, random forest, and support vector regression. JAZ. 2024:118–27. doi: 10.53555/jaz.v45is4.4164

[19] Abubakar MS, Aremu KO, Aphane M, Amusa LB. A QSPR analysis of physical properties of antituberculosis drugs using neighbourhood degree-based topological indices and support vector regression. Heliyon. 2024;10(7):e28260. doi: 10.1016/j.heliyon.2024.e28260 38571658 PMC10987931

[20] Shi X, Kosari S, Ghods M, Kheirkhahan N. Innovative approaches in QSPR modelling using topological indices for the development of cancer treatments. PLoS One. 2025;20(2):e0317507. doi: 10.1371/journal.pone.0317507 39982891 PMC11844895

[21] Kekana T, Aremu KO, Aphane M. Exploring a novel approach for computing topological descriptors of graphene structure using neighborhood multiple M-polynomial. Front Appl Math Stat. 2025;10:1508134. doi: 10.22052/IJMC.2024.253190.1733

[22] Search and Share Chemistry; 2021. http://www.chemspider.com/AboutUs.aspx

