# Supplementary material for: M-polynomial driven machine learning models for predicting physicochemical properties of antibiotics
Source: PLoS One. 2025 Dec 11;20(12):e0338093. doi: 10.1371/journal.pone.0338093 (PMC12724536; doi:10.1371/journal.pone.0338093)
Supplement: S1 Table — Available at: https://doi.org/10.6084/m9.figshare.30069574. (PDF) [file pone.0338093.s001.pdf]

Table S1. The Topological Indices and M-Polynomials for the drugs.

**chemical formulas of the drugs**	**M_1(G)**	**M_2(G)**	**H(G)**	**HM(G)**	**F(G)**	**R(G)**	**SCI (G)**	**GA(G)**	***ABC(G)***
$C_{10}H_{11}N_3O_3S$	88	100	7.51904	440	240	7.971163678	8.205562314	17.08276624	13.20292935
$C_{12}H_{17}N_3O_4S$	102	123	9.033333	516	270	9.48527	9.684977	20.21232375	15.07267
$C_{16}H_{19}N_3O_4S$	132	161	10.58571	692	370	11.23689	11.68269	24.76165886	18.86443
$C_{17}H_{18}FN_3O_3$	134	164	11.1666	682	354	11.55855065	12.23812228	26.26206059	19.20592137
$C_{16}H_{19}N_3O_5S$	137	165	10.95238	715	385	11.70565	12.11609	25.56707196	19.72137
$C_{16}H_{17}N_3O_4S$	128	155	10.9	650	340	11.41359	11.85752	25.07492781	18.67715
$C_{18}H_{37}N_5O_9$	168	202	14.26667	856	452	15.08183	15.49817	32.53096387	24.51235
$C_{18}H_{20}FN_3O_4$	146	180	11. 3333	754	394	12.37991785	13.05461886	27.99411139	20.75803431
$C_{21}H_{43}N_5O_7$	175	212	14.55238	905	481	15.34799	15.87614	33.49447857	25.17886
$C_{17}H_{25}N_3O_5S$	142	175	11.4	742	392	12.14564	12.58226	26.67300402	20.29822
$C_{21}H_{24}FN_3O_4$	166	207	13.5333	858	444	13.9904023	14.88185821	32.14425732	23.32724172
$C_{22}H_{43}N_5O_{13}$	206	247	17.83333	1046	552	18.85188	19.24748	40.18561962	30.26666
$C_{21}H_{39}N_7O_{12}$	209	251	17.61905	1079	577	18.77275	19.12545	39.88118512	30.44485
$C_{22}H_{24}N_2O_8$	184	237	13.8904	1006	532	14.86670828	15.50750685	33.25510119	25.27907326
$C_{23}H_{27}N_3O_7$	188	239	14.3238	1018	540	15.33303477	15.97986465	34.17428478	26.06706027
$C_{38}H_{72}N_2O_{12}$	269	323	21.70952	1415	769	23.35155	23.86527	49.86466262	38.81461
$C_{37}H_{67}NO_{13}$	270	324	21.64286	1426	778	23.42552	23.82631	49.62828719	39.00204
$C_{38}H_{69}NO_{13}$	274	330	22.24286	1446	786	23.98618	24.36469	50.71390527	39.55023
$C_{29}H_{39}N_5O_8$	234	289	17. 7619	1256	678	19.32201664	19.97964281	42.32250692	32.97672685
