# Supplementary material for: M-polynomial driven machine learning models for predicting physicochemical properties of antibiotics
Source: PLoS One. 2025 Dec 11;20(12):e0338093. doi: 10.1371/journal.pone.0338093 (PMC12724536; doi:10.1371/journal.pone.0338093)
Supplement: S2 Table — Available at: https://doi.org/10.6084/m9.figshare.30069577. (PDF) [file pone.0338093.s002.pdf]

Table S2. Prediction of MV and MW Properties Using Three Regression Models and Advanced Machine Learning Techniques.

chemical formulas of the drugs	Actual MV	SVR-Basic	SVR-Tuned	Random-Forest	Actual MW	SVR-Basic	SVR-Tuned	Random-Forest
$C_{10}H_{11}N_3O_3S$	173.1	289.999658	157.1839536	231.845	253.28	456.13515	229.8403778	347.3248
$C_{12}H_{17}N_3O_4S$	183.9	289.4371383	150.2347167	240.023	299.35	454.8424387	279.436745	343.877
$C_{16}H_{19}N_3O_4S$	224	288.7813362	244.0695676	231.759	331.34	453.7211928	360.6292418	340.06
$C_{17}H_{18}FN_3O_3$	226.8	288.5848021	226.6003071	228.628	347.4	453.8629234	347.1997041	356.3996
$C_{16}H_{19}N_3O_5S$	231.3	288.4438007	216.7200153	233.755	349.4	455.9863199	349.2002842	349.3762
$C_{16}H_{17}N_3O_4S$	236.2	288.5306365	225.019118	234.489	361.4	453.9090737	376.4616578	358.3194
$C_{18}H_{37}N_5O_9$	239.3	288.415941	229.0225271	235.939	365.4	454.0551091	351.8546161	366.561
$C_{18}H_{20}FN_3O_4$	268.9	289.280008	270.199536	252.714	383.5	453.5800378	368.2060082	368.0766
$C_{21}H_{43}N_5O_7$	271.1	291.1822591	270.8998454	279.369	401.4	454.7576379	445.9440802	437.377
$C_{17}H_{25}N_3O_5S$	285	290.6974993	300.7247814	265.879	444.4	456.4011657	460.3219162	446.166
$C_{21}H_{24}FN_3O_4$	293.5	293.5	293.3000187	318.164	457.5	457.5000001	475.0604325	476.782
$C_{22}H_{43}N_5O_{13}$	294.6	291.666057	297.1656938	298.162	467.5	456.888523	464.4533146	485.914
$C_{21}H_{39}N_7O_{12}$	305.9	290.9780429	313.3451077	271.26	477.6	455.8900423	477.3996493	452.7872
$C_{22}H_{24}N_2O_8$	363.9	294.3376439	380.8444016	354.589	581.6	461.4369704	581.3440019	588.929
$C_{23}H_{27}N_3O_7$	366.9	293.0583225	376.7552838	392.838	585.6	461.0989425	585.8002686	583.083
$C_{38}H_{72}N_2O_{12}$	402.5	295.2648419	426.9455895	392.214	585.6	461.8252042	599.5174436	602.591
$C_{37}H_{67}NO_{13}$	607.2	296.7843507	607.399882	604.388	733.9	462.2321046	729.7560341	728.869
$C_{38}H_{69}NO_{13}$	631.9	296.7062421	624.1832482	615.981	748	461.9876653	749.3887029	731.026
$C_{29}H_{39}N_5O_8$	632.7	296.7495282	622.9699295	617.377	749	462.2255222	731.9138871	729.692
