# Supplementary material for: M-polynomial driven machine learning models for predicting physicochemical properties of antibiotics
Source: PLoS One. 2025 Dec 11;20(12):e0338093. doi: 10.1371/journal.pone.0338093 (PMC12724536; doi:10.1371/journal.pone.0338093)
Supplement: S3 Table — Available at: https://doi.org/10.6084/m9.figshare.30069580. (PDF) [file pone.0338093.s003.pdf]

Table S3. Prediction of PO Using three Regression Models and Advanced Machine Learning Techniques.

chemical formulas of the drugs	Actual PO	SVR-Basic	SVR-Tuned	Random-Forest
$C_{10}H_{11}N_3O_3S$	24.8	43.61624539	24.84156972	33.967
$C_{12}H_{17}N_3O_4S$	28.8	41.97795538	28.86121767	33.967
$C_{16}H_{19}N_3O_4S$	33	40.8797165	33.0103691	33.881
$C_{17}H_{18}FN_3O_3$	35.4	40.83283928	35.44484653	35.485
$C_{16}H_{19}N_3O_5S$	35.7	40.85757145	35.66474522	35.354
$C_{16}H_{17}N_3O_4S$	36.1	41.22229318	36.10582725	36.328
$C_{18}H_{37}N_5O_9$	36.3	40.87906293	36.31016892	35.984
$C_{18}H_{20}FN_3O_4$	38.4	41.18177974	38.39006499	38.057
$C_{21}H_{43}N_5O_7$	40.4	43.13619103	40.30757547	42.84
$C_{17}H_{25}N_3O_5S$	43.2	45.26458985	43.22258062	44.387
$C_{21}H_{24}FN_3O_4$	44.3	43.47242123	44.28904056	44.02
$C_{22}H_{43}N_5O_{13}$	46	45.90000001	45.98973108	46.403
$C_{21}H_{39}N_7O_{12}$	48	47.89999998	48.00940824	48.861
$C_{22}H_{24}N_2O_8$	48.6	44.3832891	48.58968496	46.579
$C_{23}H_{27}N_3O_7$	53.5	47.89965397	53.47294136	51.735
$C_{38}H_{72}N_2O_{12}$	60	49.30190689	59.9781289	59.335
$C_{37}H_{67}NO_{13}$	75	49.95875449	75.01019286	74.839
$C_{38}H_{69}NO_{13}$	76.9	49.81423421	76.90333774	76.09
$C_{29}H_{39}N_5O_8$	78.3	49.87377769	78.30402857	76.474
