# Supplementary material for: M-polynomial driven machine learning models for predicting physicochemical properties of antibiotics
Source: PLoS One. 2025 Dec 11;20(12):e0338093. doi: 10.1371/journal.pone.0338093 (PMC12724536; doi:10.1371/journal.pone.0338093)
Supplement: S4 Table — Available at: https://doi.org/10.6084/m9.figshare.30069583. (PDF) [file pone.0338093.s004.pdf]

Table S4. Comparison with the Linear Regression Baseline.

Variable	Model	MAE	RMSE	R^2
COM	Linear Regression	50	70	0.95
	SVR Tuned	0.88	1.26	0.99998
	Random Forest	34.19	61.38	0.9444
MR	Linear Regression	5	6	0.92
	SVR Tuned	0.059	0.085	0.999995
	Random Forest	3.46	6.61	0.9711
MV	Linear Regression	10	12	0.93
	SVR Tuned	10.64	13.91	0.99017
	Random Forest	16.74	23.58	0.9718
MW	Linear Regression	8	10	0.94
	SVR Tuned	12.38	17.03	0.98703
	Random Forest	18.13	27.99	0.9649
PO	Linear Regression	1	2	0.91
	SVR Tuned	0.023	0.032	0.999996
	Random Forest	1.52	2.63	0.9708
