# Supplementary material for: M-polynomial driven machine learning models for predicting physicochemical properties of antibiotics
Source: PLoS One. 2025 Dec 11;20(12):e0338093. doi: 10.1371/journal.pone.0338093 (PMC12724536; doi:10.1371/journal.pone.0338093)
Supplement: S5 Table — Available at: https://doi.org/10.6084/m9.figshare.30069586. (PDF) [file pone.0338093.s005.pdf]

Table S5. Prediction of MV and MW values of test data using various algorithms.

chemical formulas of the drugs	Actual MV	SVR-Basic	SVR-Tuned	Random-Forest	Actual MW	SVR-Basic	SVR-Tuned	Random-Forest
$C_{18}H_{33}CLN_2O_5S$	88.4	293.2351116	103.7924972	312.012	138.06	457.7938521	75.71731515	347.3248
$C_{18}H_{34}N_2O_6S$	117.9	292.0069134	101.7675452	231.845	171.15	457.4131746	149.8290914	347.3248
$C_{16}H_{20}FN_3O_4$	172.4	291.1532345	164.6546362	243.634	247.27	456.201092	202.9457048	347.3248
$C_{48}H_{62}N_4O_{12}$	208.8	289.8208927	197.3445832	231.845	323.13	454.3562191	268.1511423	352.9656
$C_{46}H_{62}N_4O_{11}$	235.6	289.4618267	225.1082501	242.482	337.35	453.7235803	359.2646226	348.7836
$C_8H_{13}N_3O_4S$	259	291.3311006	274.3888503	338.669	370.3	453.980209	410.2429855	364.3616
$C_6H_9N_3O_3$	313.3	291.1117827	313.9674249	324.451	406.5	453.6877075	382.7028749	369.5608
$C_{11}H_{12}CL_2N_2O_5$	327.2	291.378446	324.354071	326.366	425	453.6721042	382.9307023	368.5578
$C_{17}H_{15}FN_6O_3$	611.7	295.4184951	643.6452389	532.921	822.9	459.4709979	839.93112	685.139
$C_3H_7O_4P$	632.4	294.4679678	682.9491514	532.921	847	458.7254099	924.4017678	686.582
