# Supplementary material for: M-polynomial driven machine learning models for predicting physicochemical properties of antibiotics
Source: PLoS One. 2025 Dec 11;20(12):e0338093. doi: 10.1371/journal.pone.0338093 (PMC12724536; doi:10.1371/journal.pone.0338093)
Supplement: S6 Table — Available at: https://doi.org/10.6084/m9.figshare.30069589. (PDF) [file pone.0338093.s006.pdf]

**Table S6.** Prediction of PO Property of Test Data Using Various Algorithms.

| chemical formulas of the drugs | Actual PO | SVR-Basic | SVR-Tuned | Random-Forest |
|---|---|---|---|---|
| $C_{18}H_{33}CLN_2O_5S$ | 10.1 | 45.93338883 | 10.20138039 | 33.967 |
| $C_{18}H_{34}N_2O_6S$ | 16.2 | 45.5387534 | 16.34619194 | 33.967 |
| $C_{16}H_{20}FN_3O_4$ | 23.4 | 44.21362328 | 23.44495103 | 33.967 |
| $C_{48}H_{62}N_4O_{12}$ | 28.8 | 42.97248523 | 28.85128288 | 33.967 |
| $C_{46}H_{62}N_4O_{11}$ | 32.9 | 41.13070462 | 32.91082226 | 34 |
| $C_8H_{13}N_3O_4S$ | 37.1 | 41.52147881 | 37.08323996 | 35.964 |
| $C_6H_9N_3O_3$ | 41.5 | 41.39250439 | 41.52632578 | 38.583 |
| $C_{11}H_{12}CL_2N_2O_5$ | 42.8 | 41.47500225 | 42.78923276 | 39.009 |
| $C_{17}H_{15}FN_6O_3$ | 84.5 | 47.32633847 | 84.33259424 | 74.268 |
| $C_3H_7O_4P$ | 88.4 | 46.17693339 | 88.23915769 | 74.268 |
