# Supplementary material for: M-polynomial driven machine learning models for predicting physicochemical properties of antibiotics
Source: PLoS One. 2025 Dec 11;20(12):e0338093. doi: 10.1371/journal.pone.0338093 (PMC12724536; doi:10.1371/journal.pone.0338093)
Supplement: S7 Table — Available at: https://doi.org/10.6084/m9.figshare.30069598. (PDF) [file pone.0338093.s007.pdf]

Table S7. Performance Analysis of Advanced ML Models on the Test Set Based on the R^2 Metric.

Models	COM	MR	MV	MW	PO
**SVR-Basic**	-3.67492	-1.63262	-0.79581	-1.41822	-1.08184
**SVR -Tuned**	**0.808577**	**0.999964**	**0.878933**	**0.928748**	**0.999945**
**Random- Forest**	0.05355	0.527315	0.414771	0.553608	0.546385
