# Supplementary material for: M-polynomial driven machine learning models for predicting physicochemical properties of antibiotics
Source: PLoS One. 2025 Dec 11;20(12):e0338093. doi: 10.1371/journal.pone.0338093 (PMC12724536; doi:10.1371/journal.pone.0338093)
Supplement: S8 Table — Available at: https://doi.org/10.6084/m9.figshare.30069604. (PDF) [file pone.0338093.s008.pdf]

Table S8. Performance Analysis of Advanced ML Models on the Test Set Based on the MSE Metric.

Models	COM	MR	MV	MW	PO
**SVR-Basic**	93176.65	1075.26	6264.686	17080.18	133.9703
**SVR -Tuned**	3815.289	0.014795	422.3426	503.2598	0.003535
**Random- Forest**	18863.86	193.062	2041.577	3152.924	29.19094
