# Supplementary material for: M-polynomial driven machine learning models for predicting physicochemical properties of antibiotics
Source: PLoS One. 2025 Dec 11;20(12):e0338093. doi: 10.1371/journal.pone.0338093 (PMC12724536; doi:10.1371/journal.pone.0338093)
Supplement: S9 Table — Available at: https://doi.org/10.6084/m9.figshare.30069607. (PDF) [file pone.0338093.s009.pdf]

Table S9. Performance Analysis of Advanced ML Models on the Test Set Based on the RMSA Metric.

Models	COM	MR	MV	MW	PO
**SVR-Basic**	305.2485	32.79115	79.14977	130.6912	11.57455
**SVR -Tuned**	61.76803	0.121635	20.55097	22.43345	0.059452
**Random- Forest**	137.3458	13.89468	45.18381	56.15091	5.402864
