# Supplementary material for: M-polynomial driven machine learning models for predicting physicochemical properties of antibiotics
Source: PLoS One. 2025 Dec 11;20(12):e0338093. doi: 10.1371/journal.pone.0338093 (PMC12724536; doi:10.1371/journal.pone.0338093)
Supplement: S10 Table — Available at: https://doi.org/10.6084/m9.figshare.30069610. (PDF) [file pone.0338093.s010.pdf]

Table S10. Performance Analysis of Advanced ML Models on the Test Set Based on the MAE Metric.

Models	COM	MR	MV	MW	PO
**SVR-Basic**	270.5834	25.9182	60.76406	105.7074	8.889493
**SVR -Tuned**	47.96403	0.095869	18.1878	18.51706	0.051543
**Random- Forest**	114.91	11.1275	42.15725	48.26675	4.2635
