# Supplementary material for: M-polynomial driven machine learning models for predicting physicochemical properties of antibiotics
Source: PLoS One. 2025 Dec 11;20(12):e0338093. doi: 10.1371/journal.pone.0338093 (PMC12724536; doi:10.1371/journal.pone.0338093)
Supplement: Attachment — Submitted filename: Response to Reviewers.docx [file pone.0338093.s012.docx]

PONE-D-25-38995

M-Polynomial Driven Machine Learning Models for Predicting Physicochemical Properties of Antibiotics

PLOS ONE

Dear Dr. Ghods,

Thank you for submitting your manuscript to PLOS ONE. After careful consideration, we feel that it has merit but does not fully meet PLOS ONE’s publication criteria as it currently stands. Therefore, we invite you to submit a revised version of the manuscript that addresses the points raised during the review process.

Please submit your revised manuscript by Oct 02 2025 11:59PM. If you will need more time than this to complete your revisions, please reply to this message or contact the journal office at [plosone@plos.org](mailto:plosone@plos.org). When you're ready to submit your revision, log on to https://www.editorialmanager.com/pone/ and select the 'Submissions Needing Revision' folder to locate your manuscript file.

Please include the following items when submitting your revised manuscript:

- A rebuttal letter that responds to each point raised by the academic editor and reviewer(s). You should upload this letter as a separate file labeled 'Response to Reviewers'.
- A marked-up copy of your manuscript that highlights changes made to the original version. You should upload this as a separate file labeled 'Revised Manuscript with Track Changes'.
- An unmarked version of your revised paper without tracked changes. You should upload this as a separate file labeled 'Manuscript'.

If you would like to make changes to your financial disclosure, please include your updated statement in your cover letter. Guidelines for resubmitting your figure files are available below the reviewer comments at the end of this letter.

If applicable, we recommend that you deposit your laboratory protocols in protocols.io to enhance the reproducibility of your results. Protocols.io assigns your protocol its own identifier (DOI) so that it can be cited independently in the future. For instructions see: <https://journals.plos.org/plosone/s/submission-guidelines#loc-laboratory-protocols>. Additionally, PLOS ONE offers an option for publishing peer-reviewed Lab Protocol articles, which describe protocols hosted on protocols.io. Read more information on sharing protocols at <https://plos.org/protocols?utm_medium=editorial-email&utm_source=authorletters&utm_campaign=protocols>.

We look forward to receiving your revised manuscript.

Kind regards,

Muhammad Ahsan, Ph.D.
Academic Editor
PLOS ONE

Journal Requirements:

When submitting your revision, we need you to address these additional requirements.

1. Please ensure that your manuscript meets PLOS ONE's style requirements, including those for file naming. The PLOS ONE style templates can be found at

https://journals.plos.org/plosone/s/file?id=wjVg/PLOSOne_formatting_sample_main_body.pdf and

<https://journals.plos.org/plosone/s/file?id=ba62/PLOSOne_formatting_sample_title_authors_affiliations.pdf>

**Response:** We have carefully reviewed our manuscript and ensured that it fully complies with PLOS ONE's style requirements, including proper file naming. All necessary adjustments have been made according to the provided style templates.

2. Please update your submission to use the PLOS LaTeX template. The template and more information on our requirements for LaTeX submissions can be found at <http://journals.plos.org/plosone/s/latex>.

**Response:** We have updated our submission to use the PLOS LaTeX template. All necessary adjustments have been made in accordance with the provided guidelines.

3. Please note that PLOS One has specific guidelines on code sharing for submissions in which author-generated code underpins the findings in the manuscript. In these cases, we expect all author-generated code to be made available without restrictions upon publication of the work. Please review our guidelines at https://journals.plos.org/plosone/s/materials-and-software-sharing#loc-sharing-code and ensure that your code is shared in a way that follows best practice and facilitates reproducibility and reuse.

Response: We have uploaded all author-generated code to Figshare, ensuring that all code will be available without any restrictions upon publication, in full compliance with PLOS ONE’s code-sharing guidelines.

4. Thank you for stating the following financial disclosure:
“This work was supported the National Natural Science Foundation of China under grant 62332006, 62172302.”

Please state what role the funders took in the study. If the funders had no role, please state: "The funders had no role in study design, data collection and analysis, decision to publish, or preparation of the manuscript."
If this statement is not correct you must amend it as needed.
Please include this amended Role of Funder statement in your cover letter; we will change the online submission form on your behalf.

**Response:** We have included the **Funding Statement** and **Author Contributions** in the cover letter. Specifically, the Funding Statement clarifies the role of the funder: Xin Li was responsible for funding acquisition, conceptualization, investigation, and preparation and review of the manuscript. All other authors’ contributions are clearly detailed in the Author Contributions section.

**Funding Statement:** This work was supported by the National Natural Science Foundation of China under grant numbers 62332006 and 62172302. Xin Li was responsible for funding acquisition, conceptualization, investigation, original draft preparation, and review and editing of the manuscript.

**Author Contributions**

- **Xin Li:** Funding acquisition, Conceptualization, Investigation, Writing–original draft, Writing–review and editing.
- **Masoud Ghods:** Data curation, Methodology, Supervision, Writing–original draft.
- **Negar Kheirkhahan:** Formal Analysis, Software, Project administration, Validation, Methodology, Writing–original draft.
- **Jana Shafi:** Validation, Visualization, Resources, Writing–review and editing.

5. Please ensure that you refer to Figure 1 in your text as, if accepted, production will need this reference to link the reader to the figure.

**Response:** We have ensured that **Fig 1** is properly cited in the text.

**Comments to the Author**

1. Is the manuscript technically sound, and do the data support the conclusions?

The manuscript must describe a technically sound piece of scientific research with data that supports the conclusions. Experiments must have been conducted rigorously, with appropriate controls, replication, and sample sizes. The conclusions must be drawn appropriately based on the data presented.

Reviewer #1: Yes

Reviewer #2: Yes

2. Has the statistical analysis been performed appropriately and rigorously?

Reviewer #1: Yes

Reviewer #2: N/A

3. Have the authors made all data underlying the findings in their manuscript fully available?

The [PLOS Data policy](http://www.plosone.org/static/policies.action#sharing) requires authors to make all data underlying the findings described in their manuscript fully available without restriction, with rare exception (please refer to the Data Availability Statement in the manuscript PDF file). The data should be provided as part of the manuscript or its supporting information, or deposited to a public repository. For example, in addition to summary statistics, the data points behind means, medians and variance measures should be available. If there are restrictions on publicly sharing data—e.g. participant privacy or use of data from a third party—those must be specified.

Reviewer #1: Yes

Reviewer #2: Yes

4. Is the manuscript presented in an intelligible fashion and written in standard English?

PLOS ONE does not copyedit accepted manuscripts, so the language in submitted articles must be clear, correct, and unambiguous. Any typographical or grammatical errors should be corrected at revision, so please note any specific errors here.

Reviewer #1: Yes

Reviewer #2: Yes

5. Review Comments to the Author

Please use the space provided to explain your answers to the questions above. You may also include additional comments for the author, including concerns about dual publication, research ethics, or publication ethics. (Please upload your review as an attachment if it exceeds 20,000 characters)

**Reviewer #1:**

1) The abstract is not expressing the novelty of the proposed approach. The whole abstract is not impressive and needs to be rewritten. Should focus on what is problem, why it is important to be solved.
How it is solved and what are findings.

**Response:** We sincerely thank the reviewer for the valuable comments. In response to the suggestions, the abstract has been completely revised to clearly highlight the novelty and significance of our proposed approach.

The revised abstract now explicitly states:

1. **The problem and its importance:** Accurate prediction of physicochemical properties of drug compounds, particularly for antibiotic therapies, and its relevance to effective drug development.
2. **The proposed method:** Use of advanced machine learning techniques, including SVR-Basic, SVR-Tuned, and Random Forest, applied to both known and previously unseen drug samples.
3. **Novelty:** Integration of feature importance analysis and ablation studies to systematically evaluate the contribution of each molecular descriptor, providing deeper insights into model robustness and key factors affecting predictive accuracy.
4. **Findings:** The approach demonstrates improved predictive accuracy compared to prior studies and offers a robust framework for real-world drug development.

We believe that the revised abstract now fully addresses the reviewer’s concerns by emphasizing the **problem, significance, methodology, novelty, and key findings** in a concise and clear manner.

2) Introduction section missing the novelty of the proposed approach; hence, the author is suggested to revise the introduction section.

**Response:** In this study, we present several innovative aspects in QSPR modeling that go beyond previous approaches. First, in addition to using topological indices and M-Polynomials as explored in prior research, we incorporate **experimental data and newly collected drug samples**, enabling a more accurate assessment of model predictive performance and generalization in real-world applications. Second, we employ **advanced machine learning models**, including basic SVR, tuned SVR, and Random Forest, which can capture nonlinear relationships between structural and physicochemical features of molecules. This allows for **more precise and reliable predictions** of drug properties, particularly for compounds used in bacterial infection treatments, compared to traditional approaches. Third, we integrate **feature importance analysis and ablation studies** to systematically evaluate the contribution of each molecular descriptor, providing deeper insights into model robustness and critical factors influencing prediction accuracy. Finally, this framework establishes a **direct connection between mathematical modeling and practical applications**, demonstrating that the predictions are not only theoretical but also valuable for the development, optimization, and clinical evaluation of new drugs.

3) There should be some lines of text between the main and sub heading. This rule should be follow in whole paper.

**Response:** We sincerely thank the reviewer for this editorial suggestion. Following the recommendation, the structure of the manuscript has been revised, and explanatory text has now been added between all main headings and subheadings to maintain logical flow and ensure that the manuscript structure complies with the journal’s formatting guidelines.

4) Figure 2 and 3 needs further illustration whereas the tables need consistent font. This rule should be followed in whole paper.

**Response:** We have revised Figures 2 and 3 to provide clearer and more detailed illustrations. In Figure 2, the comparison of machine learning models now explicitly shows performance metrics (R², MSE, MAE, RMSE) as well as the standard deviation (Std) and interquartile range (IQR) to better reflect model accuracy and generalization. In Figure 3, the error distributions are presented using both histograms and KDE plots to make variations and patterns more apparent. Additionally, all tables in the manuscript have been updated to use a consistent font. These changes ensure that the figures and tables are clearer and more informative.

5) Results section needs revision which demonstrates the training as well the testing results. In the meantime, the impacts of each consider parameter in a detailed manner which improves the validity of the approach and quality of the manuscript. Author is requested to add an ablation study which makes the manuscript more impressive.

**Response:** We sincerely thank the reviewer for the constructive feedback. Following the suggestion, the Results section has been thoroughly revised. In the revised version, we have:

1. Reported both **training and testing** results using multiple statistical metrics (R², MSE, RMSE, and MAE), as summarized in Supplementary **Tables S7–S10**,which provide a comprehensive comparison of model performance.

2. Added a detailed **feature importance analysis** (Table 11 and Figure 11) to demonstrate the contribution of each descriptor to the predictive performance of the models.

3. Included an **ablation study** (Tables 12and 13, Figures 12–16) to systematically investigate the impact of removing individual features on model accuracy. These revisions improve the clarity, validity, and overall quality of the manuscript, making the results more robust and interpretable.

These revisions improve the clarity, validity, and overall quality of the manuscript, making the results more robust and interpretable.

6) Reference section should comprise recent contributions.

**Response:** The reference section has been updated to include more recent contributions from the past few years, ensuring that the manuscript reflects the latest research developments.

7) Use proper tool for referencing.

**Response:** All references have been reformatted using an appropriate referencing tool in LaTeX to ensure consistency and correctness throughout the manuscript.

8) Overall, the formatting of the content needs the attention of the author and currently depicts poor quality formatting.

**Response:** The overall formatting of the manuscript has been thoroughly revised in LaTeX, including font sizes, headings, spacing, and alignment, to improve readability and meet standard publication quality.

9) The first page does not need to present a figure.

**Response:** The first page has been adjusted according to the reviewer’s suggestion, and all figures have been removed from it.

Reviewer #2: The manuscript presents a technically sound and well-structured application of machine learning models (SVR and Random Forest) to predict physicochemical properties of antibiotics based on M-Polynomial derived topological indices. The modeling is appropriate, and the performance evaluation is thorough and well documented.

The statistical analysis using R², MSE, RMSE, and MAE is rigorous and clearly supports the findings. The authors also provide residual and error distribution plots that add depth to the analysis.

The data used in the study appears to be fully available via public sources (ChemSpider, PubChem), and the authors provide a figshare DOI link to their code and data, ensuring reproducibility.

The manuscript is clearly written in standard scientific English, and the technical sections are intelligible to a reader with a computational background.

However, I recommend the following minor revisions before acceptance:

Clarify data preprocessing steps (normalization, outlier handling, etc.).

**Response:**
Thank you for your valuable comment. As suggested, we have revised Step 1 of the algorithm to clarify the data preprocessing procedures. In the revised version of the manuscript, we have explicitly described the handling of outliers (using the IQR method), the treatment of missing values (imputed with median values), and the normalization of features (using Min–Max scaling). These additions provide a clear explanation of how the dataset was prepared prior to model training.

Include a brief feature importance analysis, especially for the Random Forest model.

**Response:** Thank you for your valuable comment. We have updated the Methodology section to include a brief feature importance analysis for the Random Forest model. This addition highlights the most influential features in the predictions and explains how this information helps interpret the model and guide feature selection for future studies.

Provide a quantitative summary of the residuals (e.g., standard deviation or IQR).

**Response:** All requested quantitative summaries of the residuals, including mean, standard deviation, and interquartile range, have been provided in **Figure 2** and **Tables 7–9**. These figures and tables comprehensively present the residuals for all models and properties, providing a clear overview of model performance.

Add a simple baseline comparison (e.g., Linear Regression) to contextualize performance.

**Response:**
We sincerely thank the reviewer for the valuable comment. As suggested, a simple baseline comparison (e.g., Linear Regression) has been added to the appendix to provide context for model performance. To compare the performance of models predicting five variables (COM, MR, MV, MW, PO), in addition to advanced models (SVR and Random Forest), Linear Regression was considered as a simple baseline model. Key results, including MAE, RMSE, and R², are summarized in Supplementary Table S4. The data and code are available via the following link: <https://doi.org/10.6084/m9.figshare.30001105>

**Summary of Results:**

- **Linear Regression**, as a baseline model, showed reasonable performance and serves as a simple reference for comparison, but its error values are relatively higher.
- **Tuned SVR** and **Random Forest** demonstrated highly accurate and stable performance, indicating that advanced models provide better predictions.
- Untuned SVR (not shown in the table) often performed poorly, highlighting the importance of **parameter optimization**.

**Conclusion:**

Although the baseline model shows acceptable performance, **more complex models** (tuned SVR and Random Forest) provide more accurate and stable predictions and are therefore recommended for practical applications.

Consider moving some long tables to supplementary materials for readability.

**Response:** We sincerely appreciate the reviewer’s valuable suggestion regarding table length and manuscript readability. To enhance readability, detailed numerical tables have been moved to the supplementary materials, and direct links to these tables have been included in the manuscript text. This allows readers to easily access all data while keeping the main text concise and focused.

The supplementary materials include the following:

- **S1 Table.** Available at: <https://doi.org/10.6084/m9.figshare.30069574>
- **S2 Table.** Available at: <https://doi.org/10.6084/m9.figshare.30069577>
- **S3 Table.** Available at: <https://doi.org/10.6084/m9.figshare.30069580>
- **S4 Table.** Available at: <https://doi.org/10.6084/m9.figshare.30069583>
- **S5 Table.** Available at: <https://doi.org/10.6084/m9.figshare.30069586>
- **S6 Table.** Available at: <https://doi.org/10.6084/m9.figshare.30069589>
- **S7 Table.** Available at: <https://doi.org/10.6084/m9.figshare.30069598>
- **S8 Table.** Available at: <https://doi.org/10.6084/m9.figshare.30069604>
- **S9 Table.** Available at: <https://doi.org/10.6084/m9.figshare.30069607>
- **S10 Table.** Available at: <https://doi.org/10.6084/m9.figshare.30069610>
- **S1 Appendix.** Python code for predicting physicochemical properties using SVR-Basic, SVR-Tuned, and RF. Available at: <https://doi.org/10.6084/m9.figshare.28790726>

Once these clarifications are made, I believe the manuscript is suitable for publication from a technical perspective.

6. PLOS authors have the option to publish the peer review history of their article ([what does this mean?](https://journals.plos.org/plosone/s/editorial-and-peer-review-process#loc-peer-review-history)). If published, this will include your full peer review and any attached files.


If you choose “no”, your identity will remain anonymous but your review may still be made public.


**Do you want your identity to be public for this peer review?** For information about this choice, including consent withdrawal, please see our [Privacy Policy](https://www.plos.org/privacy-policy).

Reviewer #1: No

Reviewer #2: **Yes:** Osamah A.S. AL-Qalisi

 [NOTE: If reviewer comments were submitted as an attachment file, they will be attached to this email and accessible via the submission site. Please log into your account, locate the manuscript record, and check for the action link "View Attachments". If this link does not appear, there are no attachment files.]

While revising your submission, please upload your figure files to the Preflight Analysis and Conversion Engine (PACE) digital diagnostic tool, <https://pacev2.apexcovantage.com/>. PACE helps ensure that figures meet PLOS requirements. To use PACE, you must first register as a user. Registration is free. Then, login and navigate to the UPLOAD tab, where you will find detailed instructions on how to use the tool. If you encounter any issues or have any questions when using PACE, please email PLOS at [figures@plos.org](mailto:figures@plos.org). Please note that Supporting Information files do not need this step.

**Response:** Thank you for your guidance. I have uploaded all figure files to the PACE system as instructed, and all necessary checks and adjustments have been completed.

1. Please ensure that you refer to Figure 7-10 & 12-16 in your text as, if accepted, production will need this reference to link the reader to the figure.

**Response:** We thank you for your feedback on our manuscript (PONE-D-25-38995R1). We have reviewed the manuscript to ensure that all figures are explicitly cited in the text. Specifically, we have revised *Figs. 7–10* to *Figs. 7, 8, 9, and 10* and *Figs. 12–16* to *Figs. 12, 13, 14, 15, and 16*.

2. Please ensure that you refer to Figure 2-16 in your text as, if accepted, production will need this reference to link the reader to the figure.

**Response:** Thank you for your comment. We have carefully checked the manuscript and ensured that Figure 2–16 is properly referred to in the text.

3. Please include a legend for figure 2-16.

**Response:** Thank you for your comment. We have included legends for all figures in the revised manuscript.

4. Kindly update you figure files from "others" to "figure"

**Response:** Thank you for your comment. We have updated all figure files from "others" to "figure" in the revised submission.
